# Comparison of Deaths Rates for COVID-19 across Europe During the First Wave of the COVID-19 Pandemic

**DOI:** 10.3389/fpubh.2020.620416

**Published:** 2020-12-11

**Authors:** Leonardo Villani, Martin McKee, Fidelia Cascini, Walter Ricciardi, Stefania Boccia

**Affiliations:** ^1^Section of Hygiene, Department of Life Sciences and Public Health, Universitá Cattolica del Sacro Cuore, Rome, Italy; ^2^London School of Hygiene and Tropical Medicine, London, United Kingdom; ^3^Department of Woman and Child Health and Public Health—Public Health Area, Fondazione Policlinico Universitario A. Gemelli Istituto di Ricovero e Cura a Carattere Scientifico (IRCCS), Rome, Italy

**Keywords:** COVID-19, death rates, standardized mortality rate, epidemics, pandemics

## Abstract

**Background:** Europe overall suffered greatly in the early stages of the COVID-19 pandemic but the impact of different countries varied. Italy was in the forefront, but there too there were differences, with the Lombardy region the epicentre of the pandemic.

**Methods:** We report Crude Mortality Rates (CMRs) from deaths reported as due to COVID-19 and, in five countries where age-specific data are available, Standardized Mortality Rates (SMRs) in the European Union and United Kingdom.

**Results:** As of 30th August 2020, Belgium was the country with the highest cumulative CMR (86.3/100,000), but the Lombardy region reached almost double this figure (167.6/100,000), far ahead of the corresponding figure for the rest of Italy at 37.0/100,000. SMRs could be calculated for five countries (Italy, Portugal, Sweden, Germany, and Netherlands). Among them, Sweden had the highest SMR (61.6/100,000). The corresponding figures for Italy, Netherlands, Portugal and Germany were 50.2, 41.4, 15.9, and 10.1 per 100,000, respectively.

**Conclusion:** It is clear that countries within Europe have performed very differently in their responses to the COVID-19 pandemic, but the many limitations in the available data must be addressed before a definitive assessment of the reasons for these differences can be made.

## Background

Europe was the continent worst affected in the initial phase of the SARS-CoV-2 pandemic. The first cases in Europe were in Italy and deaths were soon rising rapidly in several of its northern regions, especially Lombardy (1). As they watched graphic scenes of Italian hospitals struggling to cope, European governments adopted a series of unprecedented measures to contain the spread of the virus, although with differing speed and intensity. These included restrictions on movement outside the home, rules on physical distancing, mandatory face covering in closed public settings, and introduction of elements of find, test, trace, isolate, and support systems. Even where restrictions were minimal, as in Sweden, or delayed, as in the United Kingdom, many people changed their behaviour in ways that reduced risks (2). Unlike the situation in Africa and the Americas, the initial peak of infection in Europe is now subsiding, and while some countries are seeing a resurgence associated with loosening of restrictions, it is timely to take stock of how Europe has fared in terms of deaths.

The impact of the pandemic can be measured several ways, with the two main outcomes reported being incident infection and mortality, both of which can be expressed in different ways, including trends over time and cumulatively. Both are sensitive to case definitions, which in turn are influenced by the extent of testing. Mortality rates are also affected by how the data are collected, with several countries operating separate systems collecting information from hospitals and/or long term care facilities to provide rapid information on emerging trends alongside their existing vital registration systems that allow for greater scrutiny of causes of death; definitions can vary, even within countries, in how a death from COVID-19 is defined, such as whether it is a death in someone who ever had a positive test, had one within a defined period before death, or did not have a test but had symptoms consistent with COVID-19 (3). As a consequence, excess all-cause mortality is widely viewed as the gold standard, with a recent study providing a detailed examination of 21 industrialised countries (4). It has benefits and drawbacks, as it includes deaths indirectly related to SARS-CoV-2, such as those resulting from overstretched health facilities, but it will also underestimate SARS-CoV-2 related deaths as there may be reductions in deaths from, for example, road traffic injuries.

In practice, most media and political attention has focused on reports of deaths attributed to COVID-19 in official reports. Yet their presentation often demonstrates a lack of even basic epidemiological understanding, for example as they are presented as numbers and not rates, and even less often as age-standardised rates. Given their widespread use, but recognizing their limitations, we have brought together the available data for EU countries plus the United Kingdom (UK), calculating where possible age standardized mortality rates (SMRs), and examining the situation now and cumulatively.

## Methods

We conducted an observational ecological study, comparing crude mortality rates (CMRs) and (SMRs) among EU countries and the UK. We focused on these two indicators as they best capture the trajectories of the pandemic and the impact of responses of different countries. We also examine the particular situation in Lombardy, the Italian region that was the first to report COVID-19 cases in Europe.

We obtained the absolute number of COVID-19 deaths in each EU country plus the UK as of August, 30th from the European Centre for Disease Prevention and Control (ECDC) (5). We calculated (CMRs) for COVID-19 using the daily number of deaths/100,000 resident population. We were only able to calculate SMRs for countries reporting identical age ranges (0–9, 10–19, 20–29, 30–39, 40–49, 50–59, 60–69, 70–79, >80) of COVID-19 deaths (Italy, Germany, Netherlands, Sweden, and Portugal), which we obtained from national data sources (6–10). To capture the overall burden of mortality officially attributed to COVID-19 we calculated CMRs based on cumulative deaths from 22nd February until 30th August, as reported to the ECDC and, for the five countries with age-specific data in national data sources, the age standardized cumulative figures. In the latter case, age-stratified data were available only between March 11th up to August 16th. When computing the crude mortality rates, we undertook two analyses, one including and one excluding the Lombardy region (10 million inhabitants), which was the epicentre of the Italian COVID-19 epidemics. As we were unable to use indirect standardization to compare all countries due to data limitations, we calculated the SMR by dividing the number of observed deaths in each country by the expected number of deaths. The expected deaths were estimated by multiplying the age-specific population in each country by the age-specific mortality rate of the standard population. The standard population was the total population of the EU (11). We were unable to calculate the standardized death rates in Lombardy alone, as data on age at death from COVID-19 were not publicly available.

## Results

**Figure 1:**
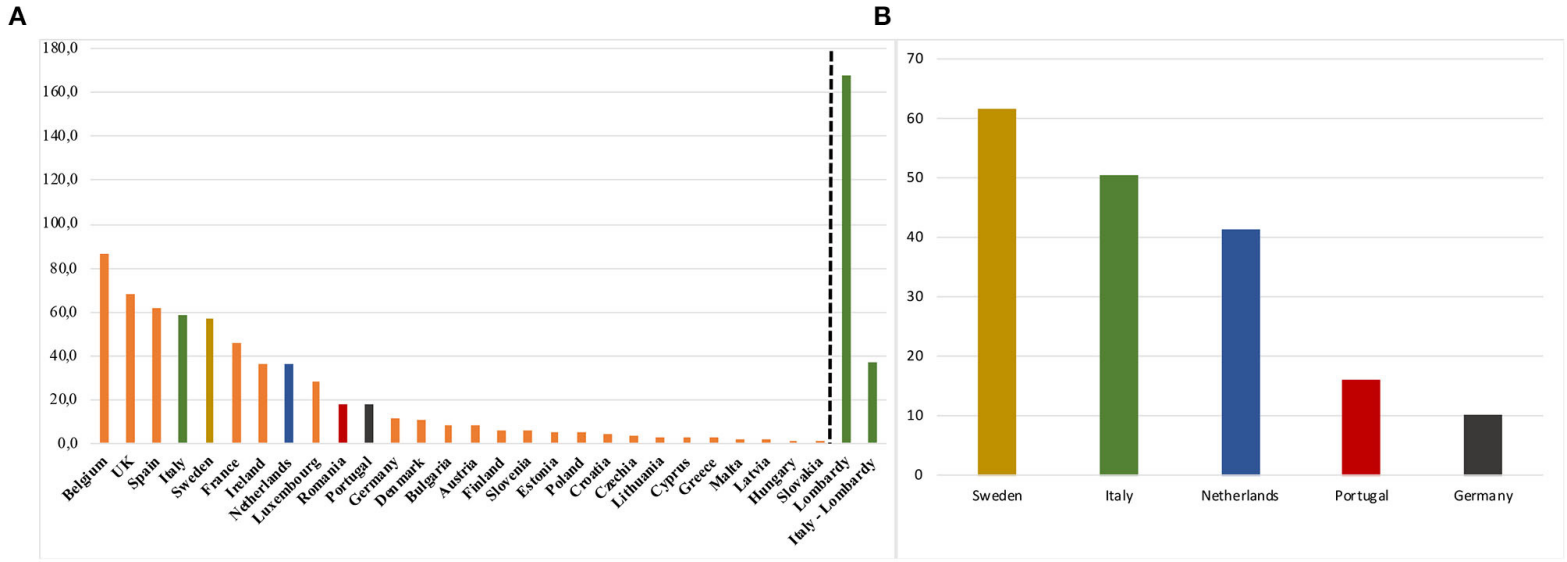
Crude Mortality Rates **(A)** for COVID-19 in 27 EU Countries plus UK, and Standardized Mortality Rates **(B)** (×100,000 inhabitants) at August, 2020.

As of 30th August, the CMR for COVID-19 varied greatly across EU countries, with Belgium reporting the highest value (86.3/100,000), followed by the UK (68.5/100,000) and Spain (62.1/100,000), while Slovakia had the lowest (0.6/100,000) (Figure 1A). When considering Lombardy region on its own, the CMR was almost twice that of Belgium, with 167.6/100,000 in Lombardy vs. 37.0/100,000 for the rest of the country (Figure 1A). Among the five countries where we could estimate age-standardised rates, Sweden reported the highest, with a SMR of 61.6/100,000, followed by Italy (50.3/100,000), Netherlands (41.4/100,000), Portugal (15.9/100,000), and Germany (10.1/100,000) (Figure 1B).

**Figure 2:**
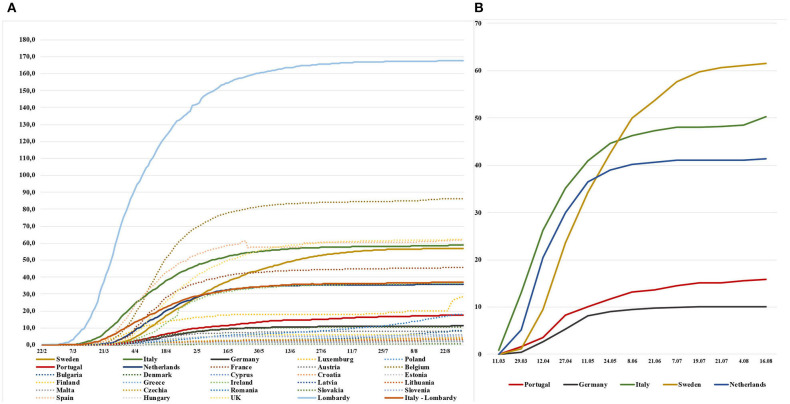
Cumulative Crude Mortality Rates (×100,000 inhabitants) for COVID-19 in 27 EU Countries plus UK **(A)**, and Cumulative Standardized Mortality Rates (×100,000 inhabitants) for selected EU Countries **(B)**.

Turning to mortality trends, Lombardy region experienced the earliest steep increase in Europe, with death rates increasing from 0.2/100,000 on 1st March to 82.6/100,000 on 1st April. The worst affected of the remaining EU countries and the UK only increased steep increases in CMRs from the beginning of April until the beginning of May, with Belgium experiencing the highest increase among the 28 countries (from 12.0/100,000 to 68.7/100,000) in this period, followed by UK (from 3.7/100,000 to 39.9/100,000) and Spain (from 17.4/100,000 to 52.9/100,000). The CMR in Sweden showed a consistent increase from the beginning of April until the end of July (from 2.8/100,000 to 56.4/100,000) and it plateaued only in the second half of August (56.9/100,000) (Figure 2A).

When looking at cumulative SMRs, the trends were similar for Italy and the Netherlands (0.9/100,000 and 0.0/100,000 on 11th March, 40.9/100,000 and 36.4/100,000 on 11th May, 48.2/100,000, 41.0/100,000 on 21st July, 50.3/100 000 and 41.4/100,000 on 16th August, respectively) where the plateau was reached at the beginning of June (Figure 2B). Similar trends, although with lower values, were also observed for Germany and Portugal (both 0.0/100,000 on 11th March, 8.1/100,000 and 10.1/100,000 on 11th May, 10.0/100,000 and 15.1/100,000 on 21st July, 10.1/100,000 and 15.9/100,000 on 16th August, respectively) with the plateau reached in the second half of May in Germany, and in the first half of June in Portugal. Reflecting the trends mentioned above, as of 16th August, Sweden has not yet reached a plateau, experiencing a constant increase (0.0/100,000 on 11th March, 32.4/100,000 on 11th May, 60.7/100,000 on 21st July and 61.6/100,000 on 16th August).

## Discussion

Before discussing our findings, it is necessary to note some limitations, not least because they have implications for policy. It seems remarkable that, in the face of a common threat that has had an enormous impact on the burden of disease in Europe, the routine hospital services (12) and the economy, governments have been unable to develop a shared understanding of what is being measured or to ensure that there are systems in place to measure it accurately and report it in a timely way. The ECDC has performed remarkably in collating and presenting the available data but it is constrained by what is collected by national and regional governments. Given that this will not be the last pandemic, this is something that should be addressed as a priority.

Our analysis does, however, have some important strengths. First, it does adjust for the age distribution of populations in some countries, rendering them more comparable, although even where we had age-specific data, the early reports from some countries had around 5–10% of missing values for age. Second, by waiting until the initial peaks had subsided, it is possible to compare the overall impact. This is a function of both the height of the peak and the time that the rate remained elevated. The importance of this can be illustrated by the situation in Lombardy. Initially there was some debate about how it had fared. Thus, despite the scenes of struggling hospitals, its death rate 30 days after the onset of the epidemic was well-below the corresponding figures in the Community of Madrid and in Brussels (41.4/100,000 in Lombardy vs. 77.1 and 48.6/100,000, respectively) (13). Yet it can now be seen that Lombardy has experienced overall the highest COVID-19 mortality rates in Europe (14). There are several possible reasons: it was the first region to be affected in Europe, at a time when there was little understanding how to manage this new illness. Lombardy adopted a hospital-centred approach, in contrast to neighbouring regions (45% of COVID-19 patients hospitalized versus 22% of other Italian regions) (15), its intensive care units were overwhelmed (16), and its nursing homes accommodated many elderly frail patients (17). The first COVID-19 clusters in the Netherlands, Germany, and Portugal started between one and two-weeks later than in Italy, by which time they had seen what was happening in Lombardy. Germany stands out from other countries. A plausible explanation relates to its much greater ICU capacity, with 29.2 beds/100,000 population in Germany vs. 8.4/100,000 in Italy, 4.2/100,000 in Portugal, and 6.4/100,000 in the Netherlands at the onset of the epidemics (16, 18). Sweden also stands out. Although it had made some recommendations about interpersonal distancing, it rejected many of the restrictions imposed elsewhere. At the time, advocates of the Swedish approach suggested that this would lead to a degree of immunity that would protect the country against subsequent waves but it is now clear that this was not the case (19).

The limitations of the data available for this analysis point to the need for future work by researchers and others. European governments and international agencies, including EUROSTAT and the WHO must find ways to collate and rapidly publish data on age at death for major causes. It is clear that the lethality of this disease increases with increasing age. Yet there is little information about whether this increase is the same everywhere. This is important information that could offer insights to inform policy but the data are lacking. More contentious, but as important, is the almost complete lack of data on mortality by ethnicity (the UK is a rare exception), so once again it is impossible to understand the scale and nature of inequalities within countries (20). Without this information, the scope for cross national learning is limited.

The COVID-19 pandemic is far from over. Already, it is clear that some countries have responded better than others. It is beyond the scope of this paper to determine why and as several countries are already experiencing a resurgence of cases, any definitive assessment would be premature. However, answers are likely to lie in three areas, political decision making, scientific advice, and health system and public health capacity (21). For now, in order to face the second wave of COVID-19, there is an urgent need to put in place systems that can provide timely, complete, and internationally comparable data (22).

## Data Availability Statement

The datasets presented in this study can be found in online repositories. The names of the repository/repositories and accession number(s) can be found in the article/supplementary material.

## Author Contributions

Material preparation and data collection were performed by LV and SB. LV and FC performed the statistical analysis. The first draft of the manuscript was written by SB and LV and MM commented on the latest version of the manuscript. WR, MM, and SB supervised the study. All authors contributed to the study conception and design. All authors read and approved the final manuscript.

## Conflict of Interest

The authors declare that the research was conducted in the absence of any commercial or financial relationships that could be construed as a potential conflict of interest.
